# Recent Advances in Nanoscale Based Electrocatalysts for Metal-Air Battery, Fuel Cell and Water-Splitting Applications: An Overview

**DOI:** 10.3390/ma15020458

**Published:** 2022-01-08

**Authors:** Tse-Wei Chen, Ganesan Anushya, Shen-Ming Chen, Palraj Kalimuthu, Vinitha Mariyappan, Pandi Gajendran, Rasu Ramachandran

**Affiliations:** 1 Department of Materials, Imperial College London, London SW7 2AZ, UK; t.chen19@imperial.ac.uk; 2 Department of Physics, S.A.V. Sahaya Thai Arts and Science (Women) College, Sahayam Nagar, Kumarapuram Road, Vadakkankulam, Tirunelveli 627116, India; anushya@savsahayathaicollege.com; 3 Electroanalysis and Bioelectrochemistry Laboratory, Department of Chemical Engineering and Biotechnology, National Taipei University of Technology, No. 1, Section 3, Chung-Hsiao East Road, Taipei 106, Taiwan; vinithavicky80@gmail.com; 4 School of Chemistry and Molecular Biosciences, University of Queensland, Brisbane 4072, Australia; p.kalimuthu@uq.edu.au; 5 Department of Chemistry, The Madura College, Vidya Nagar, Madurai 625011, India; haigaja78@yahoo.com

**Keywords:** nanoscale electrocatalyst, fabrication route, nanocomposite, specific capacity, power density, cyclic stability

## Abstract

Metal-air batteries and fuel cells are considered the most promising highly efficient energy storage systems because they possess long life cycles, high carbon monoxide (CO) tolerance, and low fuel crossover ability. The use of energy storage technology in the transport segment holds great promise for producing green and clean energy with lesser greenhouse gas (GHG) emissions. In recent years, nanoscale based electrocatalysts have shown remarkable electrocatalytic performance towards the construction of sustainable energy-related devices/applications, including fuel cells, metal-air battery and water-splitting processes. This review summarises the recent advancement in the development of nanoscale-based electrocatalysts and their energy-related electrocatalytic applications. Further, we focus on different synthetic approaches employed to fabricate the nanomaterial catalysts and also their size, shape and morphological related electrocatalytic performances. Following this, we discuss the catalytic reaction mechanism of the electrochemical energy generation process, which provides close insight to develop a more efficient catalyst. Moreover, we outline the future perspectives and challenges pertaining to the development of highly efficient nanoscale-based electrocatalysts for green energy storage technology.

## 1. Introduction

Over the years, our energy consumption has been continuously growing and mainly depends upon fossil fuel combustion, where the pollutants CO_2_, NO*_x_*, and SO*_x_* are generated as the main byproducts. Therefore, the use of fossil fuels causes a severe problem to the environment and also living beings. There is an urgent need to develop new energy resources to fulfil our energy demands more securely and sustainably. In this regard, the electrochemical conversion of hydrogen into water is considered as a fascinating clean and green energy generation methodology. To achieve this electrochemical reaction, combining water electrolysis and fuel cell technology is essential. Electrochemical reactions such as oxygen evolution reaction (OER) and hydrogen evolution reaction (HER) were achieved in a water electrolyzer, whereas hydrogen oxidation reaction (HOR) and oxygen reduction reaction (ORR) were achieved in a fuel cell [1,2,3,4].

Electrocatalysts have received much attention and interest in various applications because of their unique physical and electrochemical properties. The prime function of the electrocatalyst is to lower the overpotential of a specific electrochemical reaction that we are interested in. In particular, the size and shape of the electrocatalyst play a crucial role in electrochemical reactions because the electrocatalyst could effectively facilitate the heterogeneous electron transfer reaction between the electrode and electrolyte interface [5,6,7]. In recent years, nanotechnology has been an emerging research field in the energy application sector and therefore, several nanomaterial-based electrocatalysts have been developed with different sizes and morphologies to achieve a larger surface area, favourable spatial confinement, good mechanical strength, and superior electrocatalytic efficiency. The developed nanomaterial-based catalysts were successfully used in several applications, including electrochemical sensors [8], biosensors [9], supercapacitors [10], fuel cells [11] and batteries [12]. 

Particularly, this review focuses on designing nanomaterial-based electrocatalysts for fuel cell and metal-air battery applications due to their immense contribution to the development of next-generation clean and green energy storage systems [13,14]. Specifically, fuel cells have been expressed as viable energy storage systems. Indeed, there is a massive demand for fuel cells in the commercial industry sector due to their widespread applications in vehicles, portable electronic appliances, space probes, satellites, submarines etc., [15,16,17,18,19]. Generally, the mechanistic aspects of electrochemical reactions occur at both anode and cathode during the power generation process. In the fuel cell, the essential reactions, HOR and ORR occur at the anode and cathode, respectively. The formation of water is the final product in the overall net reaction, as described below [20].

At anode:

2H_2_ → 4H^+^ + 4e^−^ (1)

At cathode:

O_2_ + 4H^+^ + 4e^−^ → 2H_2_O (2)

Net reaction:

2H_2_ + O_2_ → 2H_2_O (3)

On the other hand, the recent research has reported that metal-air batteries performed significantly better compared to metal-ion batteries. As reported, the lithium-based metal-air batteries have a higher theoretical energy density (11,429 wh kg^−1^) compared to commercial lithium-ion batteries, which is about 30 times higher efficiency. This characteristic promotes metal-air battery as the next-generation flexible energy system [21]. A number of electrocatalysts with different metal combinations, such as CuCo_2_S_4_/NF [22], NiFe@NPC [23], CoO/CNF [24] and Pt@CoS_2_-NrGO [25] etc., have successfully been used in metal-air batteries. Each catalyst has shown unique advantages in terms of electrochemical performance. For instance, the highly active FeNx-embedded porous nitrogen-doped carbon (PNC) is one of the most representative air catalysts to construct a high-performance rechargeable metal-air battery, and the developed catalyst exhibited remarkable electrocatalytic activity (power density ~118 mW cm^−2^) towards ORR [26]. Further, the Co-N_x_/C nanorod electrocatalyst derived from the 3D zeolitic imidazole framework (ZIF) acted as the promising electrode material for battery applications, owing to its porosity, ultrahigh surface area and structural flexibility. The constructed Co-N_x_/C electrode demonstrated outstanding electrocatalytic activity towards ORR and OER compared to conventional commercial Pt/C and IrO_2_ electrodes [27]. Similarly, the hollow porous Co-N-doped carbon materials (Co-N/PCNs) developed from pyrolyzing of ZIF 8. The characterization of Co-N/PCNs catalyst indicated that it had controlled morphology and high activity towards ORR applications [28]. Furthermore, Sun et al. prepared a novel type of rare-earth-based uniformly distributed Eu_2_O_3_-Cu/NC composite catalyst by the hydrothermal route, and it showed exceptional ORR activity due to the strong synergic interaction between Eu_2_O_3_ and Cu [29]. Moreover, the water-splitting process is a vital energy generation technology, where water can decompose into oxygen and hydrogen to produce clean and sustainable energy, i.e., carbon-free highest energy density-based fuels [30,31,32]. 

This review focuses on the recent development of nanoscale-based electrocatalyst for metal-air batteries, fuel cells, and water-splitting applications. In addition, we have discussed a general reaction mechanism involved in the energy conversion process and important electrochemical parameters such as low power density, poor specific capacity, and short-term cyclic stability. Further, the catalysts fabrication routes and electrochemical performance of the fabricated catalysts for energy applications are summarized in Tables S1 and S2. Based on the fruitful discussion, nanoscale-based electrocatalyst can serve as a promising candidate for practical energy storage device applications, which are beneficial resources-in energy fields for the development of next-generation energy storage technology.

## 2. Nanoscale-Based Electrode Catalysts for Metal-Air Battery

To date, metal-air batteries have been enormously used in flexible and wearable energy devices due to their higher energy density, eco-friendly nature, good thermal stability, high ionic and electronic conductivity [33,34]. Notably, the sustainable development of recent advancements in metal-air batteries has exhibited higher theoretical energy densities than lithium-ion batteries [35]. A considerable effort has been made to investigate controllable morphology based electrocatalysts for the crucial study of cost-effective renewable energy storage technologies [36]. For example, Zhu et al. [28] synthesized a hollow porous Co-N doped carbon derived (Co-N/PCNs) electrocatalyst from metal-organic frameworks (MOFs) for ORR studies. The as-prepared Co-N/PCNs electrode materials showed significant ORR activity with a power density value of 135 mW cm^−2^ compared to the commercial Pt/C catalyst (114 mW cm^−2^). Moreover, the catalyst showed the onset potential and half-wave potential values of 0.99 V and 0.88 V, respectively, which are significantly superior to commercial Pt/C catalyst (0.98 V and 0.85 V). Further, it was mentioned that the observed catalytic response was attributed to the presence of more active sites along with the higher surface area of the catalyst. The density functional theory confirmed that Co-N/PCNs have more active sites and defects that are beneficial for O_2_ adsorption and thus attain superior catalytic performances. It was evident that the morphological characteristics of the developed catalyst greatly enhanced the catalytic performance, thereby reducing the total cost of the energy system. Recently, Wang et al. [37] developed a one-dimensional nano-rod like structured Mn_3_O_4_/NiCo_2_S_4_ nanocomposite by deposition of NiCo_2_S_4_ on Mn_3_O_4_ matrix through the sulfidation method. During the sulfidation process, a diffusion staggered interface region was observed between Mn_3_O_4_ and NiCo_2_S_4_, which effectively induced more oxygen vacancies and defects. This staggered interface has a more active centre and thus significantly enhances the catalytic performance of Mn_3_O_4_/NiCo_2_S_4_ in both ORR and OER. Further, it displayed a maximum power density of 106.26 mW cm^−2^ with excellent long-term cyclic stability (650 cycles @ 5 mA cm^−2^). 

Moreover, by the combination of quantum dots (QDs) and carbon nanotubes (CNTs), the ultra-sized iron-based NiFe_2_O_4-_QDs/CNTs electrocatalyst synthesized through the hydrothermal method, and the resulting catalyst was extensively applied in OER activity. The developed electrocatalyst exhibited an onset potential and power density of 0.9 V and 275 mW cm^−2^, respectively, with superior long term cyclic durability towards OER activity [38]. Further, Liu et al. [39] demonstrated the fabrication of high-purity and unique geometrically based Co@CoO*_x_*/HNCNT composite through in situ chemical vapour deposition (CVD) followed by hydrothermal strategies. The catalyst possesses richer edge defects and a high surface area, and these characteristics effectively promote catalytic efficiency toward ORR with good charging and discharging performance. Further, electrochemical parameters were estimated and it was found that the catalyst displayed a high open-circuit voltage (1.48 V), larger specific capacity (367.31 mAh g^−1^) and a maximum power density (3.86 mW cm^−2^). In another report, Deyab and Mele demonstrated the fabrication of polyaniline-based Zn phthalocyanine (PANI/ZnPc) by the direct solid-state mixing method and the designed catalyst was potentially utilized for ORR applications in metal-air batteries [40]. 

**Figure 1:**
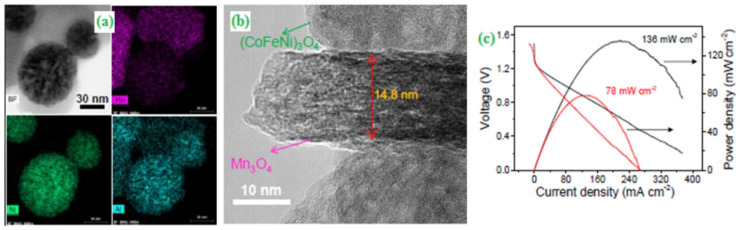
(**a**) Bright–field STEM and EDS mapping of the dealloyed AlFeCoNiMn sample for FeCoNi–based oxide nanoparticles and Mn_3_O_4_ nanosheets (**b**) HRTEM image of the Mn_3_O_4_ nanosheets in the dealloyed AlFeCoNiMn, and (**c**) Polarization and power density curves of the liquid batteries. Copyright 2020 by American Chemical Society [41].

Moreover, multi-components incorporated transition-metal oxide-based electrocatalysts have been widely employed for battery-related applications. Li et al. introduced a generic and scalable synthetic approach to produce Mn_3_O_4_-based and FeCo-based spinel oxide nanocomposites by dealloying Al-rich precursors such as Al_92_Fe_2_Co_2_Ni_2_Mn_2_ [41]. As reported, the aluminium metal has an inherent drawback in the metal-air batteries due to the formation of a passive oxide film which decreases the overall battery performance [42]. Therefore, Al metal in the precursor acts as a sacrificial element upon the incorporation of other metals. XRD data revealed that Al diffraction peaks had vanished entirely after the annealing of precursor and subsequently evolved new peaks corresponding to the Co_3_O_4_, CoFe_2_O_4_ and Mn_3_O_4_. Scanning transmission electron microscopy-energy-dispersive X-ray spectrometry (STEM-EDS) mappings technique divulged that FeCoNi-based oxide and Mn-based oxides developed in the form of a nanosphere and nanosheet, respectively (Figure 1a). The HR-TEM image indicates that Mn_3_O_4_ nanosheets exist as the elongated sphere-like nanoparticles with a size of 50 nm and a thickness estimated to be 10–20 nm (Figure 1b). Based on the polarization studies, the AlFeCoNiMn modified electrode showed a higher open-circuit potential (1.44 V) compared to the commercial Pt/C + IrO_2_ electrode (1.41 V) (Figure 1c) [41]. 

**Figure 2:**
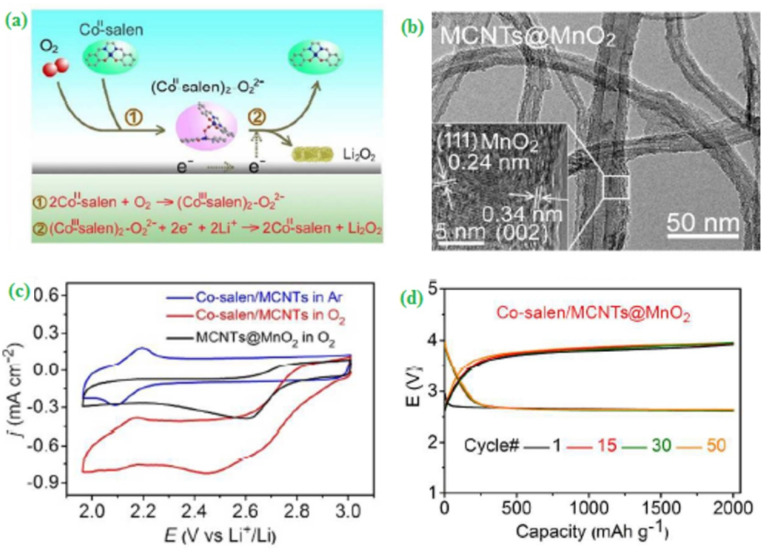
Synthesis and analysis of Co–salen/MCNTs@MnO_2_ (**a**) Schematic illustration for the reaction mechanism of the LABs with Co^II^–salen in the electrolyte during discharge and charge process, (**b**) TEM images of MCNTs@MnO_2,_ (**c**) Cyclic voltammograms obtained for ORR at a scan rate of 10 mV s^−1^ and (**d**) Discharge/charge profiles for Co–salen/MCNTs@MnO_2_ at 500 mA g^−1^ with the cut-off capacity of 2000 mAh g^−1^. Copyright 2017 by American Chemical Society [45].

Usually, semi-solid supported solid-state electrolytes play a crucial role in designing zinc-air batteries (ZABs), and they can influence various electrochemical parameters like a higher reaction rate, power output and excellent cyclic performances. Fan et al. [43] fabricated a novel porous structured and electroactive conducting polymer (Polyvinyl alcohol, PVA) based silica (PVA/SiO_2_) nanocomposite for the development of high-performance ZABs. The resulting catalyst had an excellent ionic conductivity value (57.3 mS cm^−1^) along with an excellent water retention capability. These characteristics can drastically improve the thermal and mechanical properties of the catalyst and subsequently support the improvement of the cyclic stability and power density of the ZABs. In general, the nitrogen doping process has become an essential tool to enhance surface active sites and catalytic performance of the catalysts. In this regard, Zahoor and co-workers [44] proposed a nitrogen-doped porous ultrathin nanosheet oriented MnO_2_/N-rGO composite for ORR applications. In terms of ORR activity, MnO_2_/N-rGO exhibited the onset potential at −0.05 V vs Hg/HgO and the observed specific capacity value of 5250 mAh g^−1^ at 0.2 mA cm^−2^. Further, MWCNTs integrated MnO_2_ (MWCNTs@MnO_2_) nanocomposite prepared, and subsequently, it coupled with N,N′-bis(salicylidene)ethylene diamino cobalt (II) (Co^II^-salene) to generate the electrocatalyst (Co^II^-salene/MWCNTs@MnO_2_) for ORR (Figure 2a). Based on the TEM analysis, the incorporated MWCNTs@MnO_2_ pristine nanocomposites exist in an average thickness of 2 to 3 nm (Figure 2b). The different nature of electrocatalysts and their respective redox behavior were tested through the cyclic voltammetry technique (Figure 2c). The developed catalyst was applied to investigate the ORR activity and compared with its precursors. The catalytic activity of MWCNTs@MnO_2_/Co^II^-salene was superior to other electrodes, and it delivered energy density values of 325 Wh kg^−1^ with a long cycle life (300 cycles) (Figure 2d) [45].

Diatomite has been used as one of the oxygen reversible electrocatalysts for the application of ORR and OER activity. Accordingly, viable and cost-effective porous structured CoPt-1/diatomite-C nanocomposites have been considered as a yardstick of ORR and OER. Moreover, the developed electrocatalyst was exhibited a larger current density (4.94 mA cm^−2^), lower Tafel slope (63 mV dec^−1^) and high performance (specific capacity value of 616 mAh g^−1^@10 mA cm^−2^) of ZABs applications [46]. Hao et al. [47] have recently developed nitrogen-doped porous hollow spheres that supported FeOx@N-PHCS bifunctional electrocatalyst for ORR and OER applications. To design the composite, a self-sacrifice template such as melamine-formaldehyde resin spheres were used, while for the N and C sources, the polydopamine was utilized. XRD data revealed that pristine PVA exibits a semicrysalline nature due to the presence of strong hydrogen bonding of the hydroxyl groups. However, this crystalline structure was transformed into amorphous nature upon the incorporation of SiO_2_. The authors pointed out that the structural transformation enormously improved the ionic conductivity due to the generation of a more hydrophilic domain. The fabricated FeO_x_@N-PHCS catalyst has a larger specific surface area (236 m^2^ g^−1^), numerous Fe-N_x_ active sites, fast-mass transfer process, and these characteristics offer immense catalytic activity in the performance of ORR and OER.

**Figure 3:**
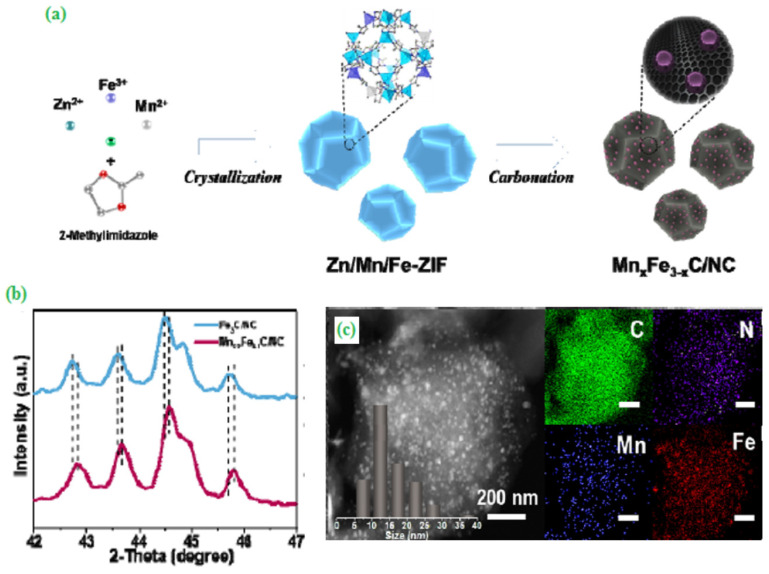
(**a**) The schematic diagram for the fabrication process of MnxFe_3-x_C/NC catalyst, (**b**) XRD patterns of Fe_3_C/NC and Mn_0.9_Fe_2.1_C/NC, (**c**) STEM image with its corresponding elemental mappings for C, N, Mn, and Fe of the Mn_0.9_Fe_2.1_C/NC catalyst. Copyright 2019 by American Chemical Society [48].

A significant effort has been made to develop a robust bi-functional electrocatalyst for ZABs application by integration of manganese-iron binary carbide nanoparticles and nitrogen-doped graphitic carbon (Mn_0.9_Fe_2.1_C/NC) as illustrated in Figure 3a. From the X-ray diffraction (XRD) analysis, Mn_0.9_Fe_2.1_C/NC exhibited a (002) graphitic carbon plane, which was confirmed by the successful incorporation of Mn into iron carbide (Fe_3_C) (Figure 3b). Interestingly, the STEM analysis divulged that Mn_0.9_Fe_2.1_C/NC appeared as the homogeneous polyhedral shape with an average particle size of ~15 nm, as shown in Figure 3c. The resulting Mn_0.9_Fe_2.1_C/NC catalyst displayed high-power density (160 mW cm^−2^) with a larger energy density (762 mWh gzn^−1^) and an impressive cyclic stability (1000 cycles) [48]. 

The Co/CoFe_2_O_4_ decorated carbon (CCFOC)-based nanocomposite synthesized by the auto combustion method, and the resulting composite electrode showed excellent electrocatalytic activity towards ORR and OER. It has been reported that the developed CCFOC had a high active surface area with more active sites, which accelerated the fast electron transfer kinetic process and consequently obtained superior ORR and OER activities. Further, the porosity of the catalyst was tuned by changing the starting material such as glycine/nitrate and cobalt nitrate/iron nitrate ratios. Further, the CCFOC electrocatalyst demonstrated a good discharge capacity (4320 mAh g^−1^@100 mA g^−1^) with long-term cyclic durability (100 cycles) [49]. Interestingly, the fabricated split cell Li-air battery was successfully applied to continuously power a commercial 2.8 V green LED bulb for more than 6 h.

**Figure 4:**
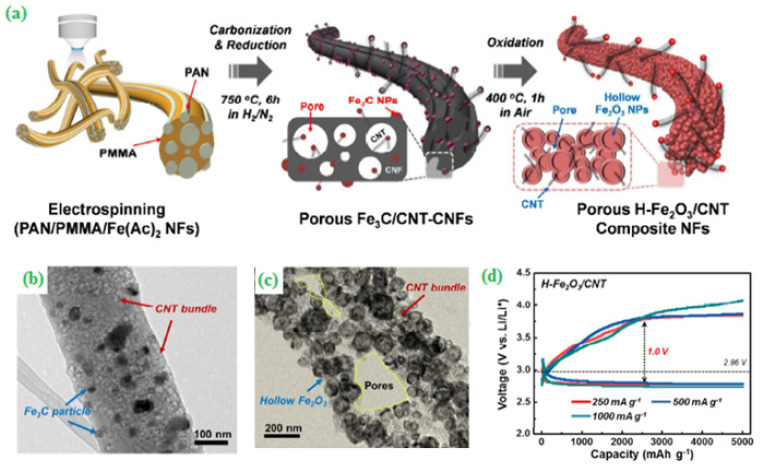
(**a**) Schematic illustration for the synthetic protocol of porous H-Fe_2_O_3_/CNT NFs, (**b**) TEM images of Fe_3_C/CNT-CNFs, (**c**) TEM images of H–Fe_2_O_3_/CNT NFs, (**d**) Comparison of overpotentials of H–Fe_2_O_3_/CNT NFs with a capacity limit of 5000 mAh g^−1^ running at different rates (250, 500 and 1000 mA g^−1^). Copyright 2018 by American Chemical Society [50].

Moreover, Jung et al. [50] fabricated and studied ORR and OER activity of hierarchically structured CNT based hollow Fe_2_O_3_ nanoparticles by the electrospinning method using polyacrylonitrile (PAN) as a precursor and poly(methyl methacrylate) (PMMA) as co-polymer (Figure 4a). The randomly mixed nano-onion H-Fe_2_O_3_/CNT NFs composite with an average particle size thickness value larger than 50 nm was analyzed by TEM (Figure 4b,c). In this connection, the constructed H-Fe_2_O_3_/CNT NFs composite showed an outstanding cell performance (specific capacity = 1000 mAh g^−1^@500 mA g^−1^) with a long life cycle (250 cycles) (Figure 4d). A multi-metal (Fe, Co, Cu and Zn) blended zeolite imidazole framework (ZIF) via a simple redox reaction route was proposed to develop a novel bifunctional electrocatalyst for ORR and OER. After the pyrolysis process, a highly porous carbon polyhedron (FC-C@NC) was obtained. The catalyst delivered a discharge capacity of 659.5 mAh g^−1^ at a current density of −4.66 mA g^−1^ with a good cyclic stability [51]. 

**Figure 5:**
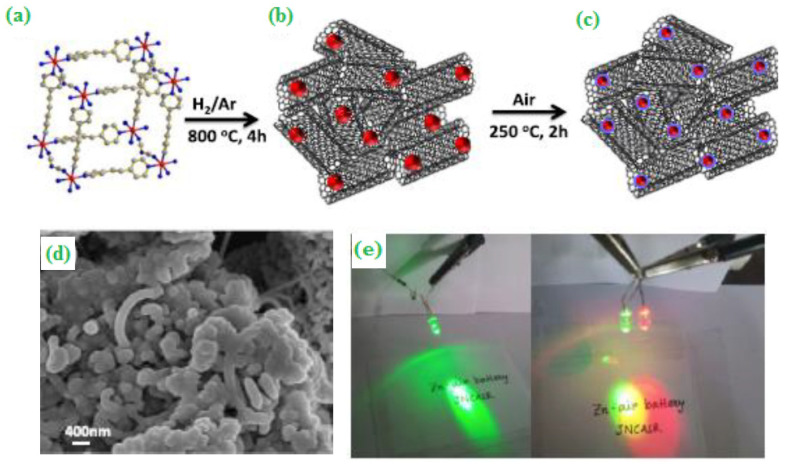
(**a**) Crystal structure of {[Co(bpe)_2_(N(CN)_2_)]·(N(CN)_2_)·(5H_2_O)}_n_ MOF (CoMOF-1), (**b**,**c**) Carbonization process and the core Co/NCNT obtained as the intermediate followed by the calcination leading to the formation of Co_3_O_4_@Co/NCNT, (**d**) FE-SEM image of Co_3_O_4_@Co/NCNT, and (**e**) Optical image showing brightly lit LEDs. Copyright 2020 by American Chemical Society [52].

A MOF-derived core-shell structured Co_3_O_4_@Co/NCNT tri-functional electrocatalyst was synthesized by the carbonization template route and the developed catalyst has shown tremendous electrocatalytic activity towards ORR and OER (Figure 5a–c). The diamond-like structured and elemental mapping regularly distributed Co, C, N and O in the Co_3_O_4_@Co/NCNT trifunctional matrix, and was analyzed by FE-SEM (Figure 5d). The constructed cells were tested and lit up by light-emitting diodes (LEDs) (Figure 5e) [52]. Further, phosphorous-based carbon dots supporting graphene (P-CD/G) nanocomposite were synthesized by a biomass-derived route, which showed an impressive power density of 157.3 mW cm^−2^. This electrocatalyst can be used as a unique tool for developing future portable and wearable electronic devices [53].

Further, it has been demonstrated that the structurally and morphologically oriented composite catalyst showed an excellent catalytic performance ensuing a larger surface area and a higher charge storage capability [54]. For instance, the corrosive resistive natured and highly porous morphological cobalt supported carbon nanotube (Co-ZIF-67/CNT) composite has shown a high structural integrity and good electrical conductivity in fuel cell and metal-air battery and alkaline exchange membrane fuel cell (AEMFC) applications [55]. Further, the Co-ZIF-67/CNT displayed the highest power density of 296 mW/cm^2^ and 60 mW/cm^2^ towards ZAB and AEMFC systems, respectively, which are comparable to the values obtained for commercial 40 wt.% Pt/C catalyst (317 mW/cm^2^ for ZAB and 64 mW/cm^2^ for AEMFC). 

The trifunctional electrocatalysts are considered as suitable materials for sustainable energy storage technologies [56]. Recently, coupling of FeM (M = Ni & Co) with nitrogen-doped porous carbon (FeM/NPC) composite was employed through a facile and scalable strategy. The developed FeM/NPC catalyst showed an excellent catalytic activity towards ORR, HER and OER [57]. The authors indicated that the observed activity ascribed to multiple factors of the FeM/NPC such as a high porous structure and surface area, excellent conductivity, hetero nitrogen atom doping, as well as close interaction between FeM and NPC. In another report, the highly efficient metal-organic framework-based N-doped carbon (MO-Co@NC) bifunctional catalyst developed through the pyrolysis of a bimetal metal-organic framework which contains Zn and Co, as the precursor. The resulting catalyst posed a highly porous structure and larger surface area, which offers an excellent catalytic response towards ORR/OER with outstanding durability [58]. It was mentioned that ORR is facilitated by the pyridinic N content, while OER is promoted by the surface contents of Co–N*x* and Co^3^^+^/Co^2^^+^. Further, the rapidly developed Mn nanomaterial-based mesoporous helical structured N-doped carbon nanotube (Mn@HNCNT) composite has been reported to act as a precious metal catalyst for ORR [59]. The catalyst was fabricated via molten-salt method at high temperature. The precursor melamine was used as carbon and nitrogen sources to generate N-doped carbon matrix, and MnCl_2_ was used to generate Mn nanoparticles. The optimized Mn@HNCNT has plenty of active sites, which tremendously improved ORR activity in metal-air battery applications. 

**Figure 6:**
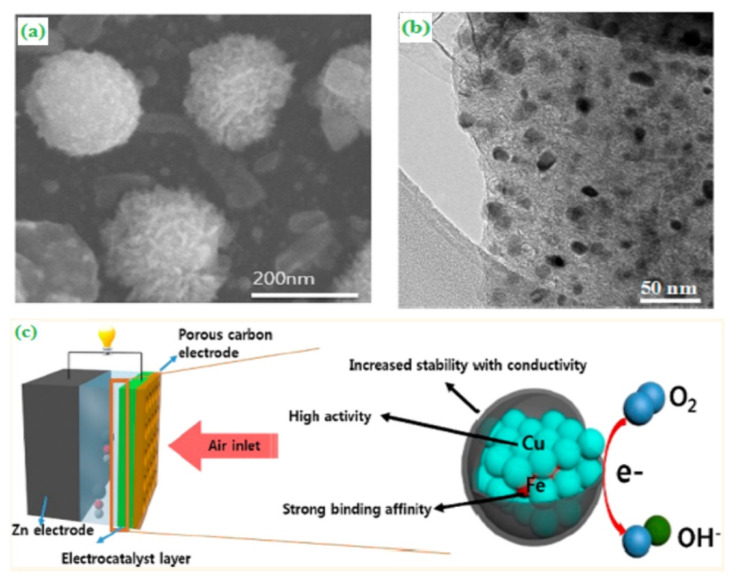
(**a**) SEM image of CuFe, (**b**) HR–TEM image of CuFe, and (**c**) schematic representation of ORR catalytic function of CuFe. Copyright 2015 by American Chemical Society [60].

The paralysis technique was employed to synthesize the noble-metal-free and nanoscale-based transition metal oxide CuFe alloy coated core-shell structured graphitic carbon electrocatalyst. The morphological structure of CuFe supported graphitic carbon was evaluated by SEM and TEM analysis. The as-prepared electrocatalyst has been displayed as nanoscale-based metal alloys, and the coated graphitic carbon looked like a ball-like structure (Figure 6a,b). The core-shell based morphological electrocatalyst has a moderate binding strength and also catalytically influenced their charge-transfer process in the rechargeable metal-air battery (Figure 6c) [60]. 

**Figure 7:**
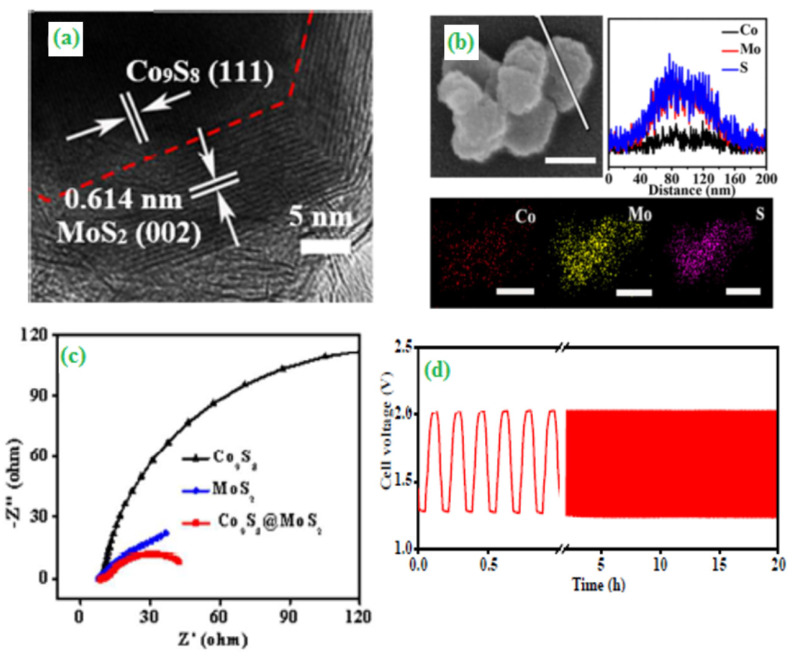
(**a**) HR-TEM image of the Co_9_S_8_@MoS_2_, (**b**) FE-SEM image (scale bar: 100 nm), corresponding cross-sectional compositional line-scan profiles and elemental mapping of the Co_9_S_8_@MoS_2_, (**c**) EIS plots recorded at −0.143 V and (**d**) Galvanostatic charge-discharge cycling at a current density of 10 mA cm^−2^. Copyright 2018 by American Chemical Society [63].

Zhao et al. designed a hierarchically arranged porous carbon nanofiber-based non-precious metal-doped nitrogen (HP-Fe-N/CNFs) catalyst via pyrolysis of polypyrrole-coated electrospun polystyrene/FeCl_3_ fibers [61]. As reported, effective doping of N and Fe was achieved in the catalyst with other important physical parameters such as high specific surface area (569.6 m^2^/g) and a large pore volume (1.00 cm^3^/g), which could promote the ORR catalytic activity under alkaline conditions. Further, the HP-Fe-N/CNFs outperforms 30 wt% Pt/C when applied in Zn-air batteries in terms of power density and long-term operational stability. Wang and co-workers [62] summarized the design and development of different morphologically oriented electrocatalysts and found that electrochemical performances enhanced upon reducing the particles size due to increasing of active surface area. The uniformly distributed core-shell morphologically based heterostructured Co_9_Se@MoS_2_ electrocatalysts were successfully synthesized using a solvothermal method followed by thermal conditions (750 °C). The electrode surface morphology and their particles diameter were analyzed through HR–TEM (Figure 7a,b). From the electrochemical impedance spectroscopic studies, the charge-transfer values were decreased and significantly promoted their electrocatalytic activity towards OER (Figure 7c). The designed core-shell structured Co_9_Se_8_@MoS_2_ catalysts exhibited better cyclic stability during the galvanostatic charge-discharge process (Figure 7d) [63]. The in-situ growth of metal-containing (Ni, Co and Pt) ceramic composite was integrated with CNT to form a catalyst. The resulting ceramic monolith composite can act as cathode electrode materials to develop anion exchange membrane fuel cell (AEMFC) applications. Moreover, in FE-SEM analysis, the metal-containing ceramic monolith showed a foam type of morphological structure with interconnected particles diameter ranging from 0.5 to 1.0 mm. The ORR and OER electrochemical activity of the resulting electrocatalyst was also assessed. The proposed cathode cell delivered a power density value of ~10 mW cm^−2^ from the polarization analysis [64].

## 3. Nanoscale Based Electrode Catalysts for Fuel Cells 

As discussed earlier, fuel cells have shown the most renewable energy storage system owing to their unique characteristics like low cost, reduced emissions and high effectiveness, which meet future global energy demands [65]. A ceramic nanocomposite fuel cell (CNFC) is a new energy system that operates at lower temperatures and uses materials from solid oxide fuel cell (SOFC) and molten carbonate fuel cell (MCFC) technologies [66,67,68,69]. Interestingly, Asghar et al. [70] developed a LiNiCuZn oxide using a slurry technique, and it has been identified as a promising substitute for CNFC applications. At a temperature of 550 °C, the as-prepared material has an outstanding performance of 1.03 W cm^−2^. The acquired value was consistent with the value reported in the literature (1.1 W cm^−2^) for a NiO-based anode. Furthermore, the microscopic analysis indicated that the particles are porous with a well connected network at the electrode. The structure of LiNiCuZn-oxide is confirmed by XRD analysis. Makharia et al. [71] proposed a method for developing Pt-alloy cathode catalysts with increased activity compared to commercial Pt/C and also the current generation of PtCo/C, with the long-term objective of 4-fold mass activity gain vs. Pt/C. At the same time, these novel catalysts must retain their mass activity for lengthy voltage cycling as well as their specific activity throughout a long time operation. 

The polymer electrolyte membrane fuel cell (PEMFC) is used as a potential renewable energy technology for transportable and stationary devices. This might be because of its cost-effectiveness, good efficiency, low-temperature operation, minimal emission and quick start up time [72]. Polyethylene terephthalate (PET) waste bottles were utilized to synthesize reduced graphene oxide (rGO) and reduced grapheneoxide/magnetic iron oxide nanocomposite (rGO/MIO) as anode and cathode, respectively. A facile approach was adopted in order to decrease production costs. This study found that employing gas diffusion layer (GDL) improved membrane electrode assembly (MEA) performance by 66% when compared to MEA without GDL at a current density of 0.8 A cm^−2^. The developed materials could be utilized as active catalyst electrodes for ORR in fuel cells with a long lifespan [73]. 

**Figure 8:**
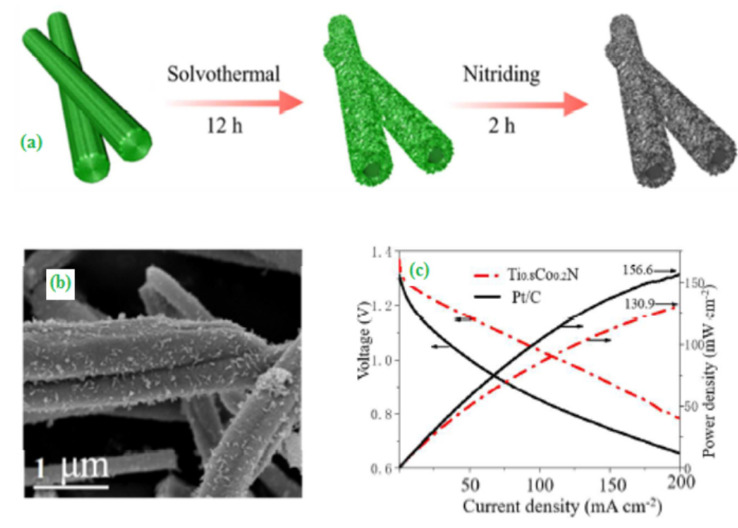
(**a**) Schematic diagram for the preparation of titanium nitrides assemblies, (**b**) SEM image of TiOSO_4_ precursors formed at 4 h reaction time, and (**c**) Polarization and power density curves for Ti_0.8_Co_0.2_N. Copyright 2018 by American Chemical Society [74].

Tian et al. [74] used a facile approach to synthesize metal nitride nanosheets through an additive/template-free hydrothermal approach followed by nitridizing titanium-based dioxides as shown in Figure 8a. The hydrolysis of precursor TiOSO_4_ yields a nanosheet structure (Figure 8b) due to the dissolution of the inner core and the redeposition of titanium-associated products on the external surface. Interestingly, it was found that TiOSO_4_ rod acts as not only the Ti source but also used as self-templates to generate a nanosheet. The as-prepared Ti_0.8_Co_0.2_N nanosheets displayed significant ORR activity in both acidic H_2_-air and alkaline Zn-air fuel cells. Consequently, the nanosheets can be used as a promising application in real alkaline metal-air fuel cells (Figure 8c). 

In the past, platinum was thought to be the most significant or key electrode to be used as an electrode in these energy devices. Nevertheless, the primary challenge of using platinum as electrode material in a commercial fuel cell is the cost, which makes it inappropriate for commercial usage. As a result, the most efficient and low-cost material to utilize as an electrode in fuel cells, such as Perovskite materials (LaCoO_3_ and LaMnO_3_) is deemed vital [75]. A general formula for perovskites is ABO_3_. It exhibits a high thermal stability with a 3–4 eV band gap [76]. Several perovskite-based materials have been used as an effective electrocatalyst for energy applications. Developing Fe-based Bi_0.5_Sr_0.5_Fe_0.95_Mo_0.05_O_3−δ_ (BSFM) perovskite electrodes has been researched effectively as a possible electrode option for practical use in solid oxide fuel cells [13,77]. Several physical and electrochemical properties (CO_2_ tolerance, phase structure, and thermal expansion behavior) of BSFM were investigated. The fabricated catalyst served as an excellent electrocatalyst for ORR and achieved the lowest polarization resistance of 0.12 Ω cm^2^ and excellent current density of 250 mA cm^2^ at a constant overpotential of 70 mV. When applying the BSFM as cathode material in the fuel cell, it achieved a maximum power density of 1.07 W cm^−2^ at 700 °C.

To develop novel nanocomposite membranes, Fe_2_TiO_5_ is functionalized with SO_3_H-containing polymers and then subsequently integrated into a sulfonated polyether ether ketone (SPEEK) matrix. The study revealed that membrane characteristics such as fluid consumption, dimensional stability and proton conductivity were greatly enhanced. Moreover, at 80 °C, poly(2-acrylamide-2-methyl-1-propane sulfonic acid)/Fe_2_TiO_5_ (S/PA@FT2) and poly(4-vinyl benzene sulfonate)/Fe_2_TiO_5_ (S/PV@FT2) exhibited maximal proton conductivity of 0.222 and 0.209 S cm^−1^, respectively. These results disclose that the prepared membranes are excellent candidates to use as proton exchange membranes in polymer electrolyte membrane fuel cells (PEMFCs) [78]. Various Pd (Palladium), Ag (Silver) and Pd-Ag (Palladium-Silver) alloy nanoparticles were synthesized individually on the rGO matrix by co reducing the corresponding metal precursors and GO with hydrazine at 60 °C. The as-prepared composites were utilized as an anode catalyst for butanol oxidation in alkali. The Pd_70_Ag_30_@rGO materials have shown an enhanced synergic catalytic activity. Furthermore, the electrode Pd_70_Ag_30_@RGO is the best since it can maintain a steady peak current even after a hundred cycles of CV operation [79]. 

**Figure 9:**
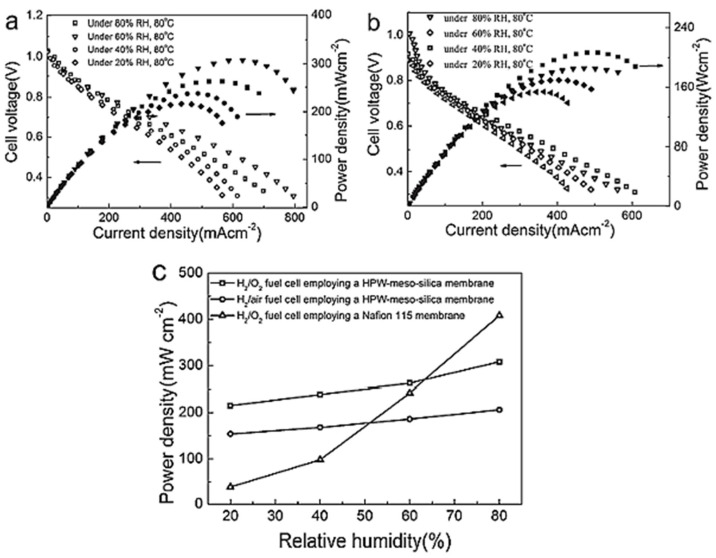
Polarization and power density of single cells developed an 80% HPW-meso-silica nanocomposite membrane in (**a**) H_2_/O_2_ and (**b**) H_2_/air at 80 °C under various relative humidity (RH) values. (**c**) The plot of the dependence on RH of the maximum power density of cells with 80% HPW-meso-silica and Nafion 115 membranes. Copyright 2011 by American Chemical Society [80].

Phosphotungstic acid, H_3_PW_12_O_40_ (HPW)-meso silica nanocomposite was used to develop a novel inorganic proton-exchange membrane that was extensively studied by Zeng et al. [80]. At 80 °C, the single-cell performance of the 80% HPW meso-silica membrane functioning in both H_2_/O_2_ and H_2_/air as a function of relative humidity (RH) is shown in Figure 9a. The cell operating in H_2_/O_2_ at 80 °C has an OCV (open circuit voltage) of 1.01V. The resulting value is near the OCV found for single cells constructed with Nafion membranes, indicating that the prepared membranes are resistant to hydrogen crossover. At 80 °C and 80 % RH, a high-power density of 308 mW cm^−1^ was achieved in H_2_/O_2_ and 206 mW cm^−2^ in H_2_/air, which is considerably higher than the values reported [81,82,83,84,85,86]. The developed nanocomposites power output might be ascribed to the proton conductivity. The significant reduction in cell voltage performance in H_2_/air at a low currents suggests that activation polarization limits performance, predominantly on the cathode side, most likely owing to a weak and not-optimized interface between the Pt/C catalyst layer and the HPW-meso silica membrane (Figure 9b). Figure 9c compares the peak power density of cells with HPW-meso-silica and Nafion 115 as a function of RH at 80 °C. Under 80% RH, the power density of the cells containing Nafion 115 was 400 mW cm^−2^, then it diminished to 38 mW cm^−2^ when the RH was reduced to 20%. Additionally, the synthesized nanocomposites can be used as active proton exchange membranes for high-temperature PEMFCs and DMFCs.Ni_0.5_Zn_0.5−x_Ce_x_-oxides (NZC oxides) [87]. 

According to XRD, the synthesized nanocomposites are present and crystalline in nature and have a multiphase structure. Moreover, the phase change does not occur as a result of the treatment of CH_4_. The quantum confinement results depicted using XRD reveal that the crystalline size of the nanocomposites declines first from 30–22 nm and subsequently increases with increasing cerium concentrations. The observed crystalline size differences might be attributed to differences in the ionic radii of Ni^+^, Zn^+^ and Ce^+^ ions. Ni_0.5_Zn_0.3_Ce_0.2_-oxides have a conductivity of 7 S cm^−1^ at 600 °C. These findings suggest that Ni_0.5_Zn_0.3_Ce_0.2_-oxides could be a suitable anode material for SOFCs.

**Figure 10:**
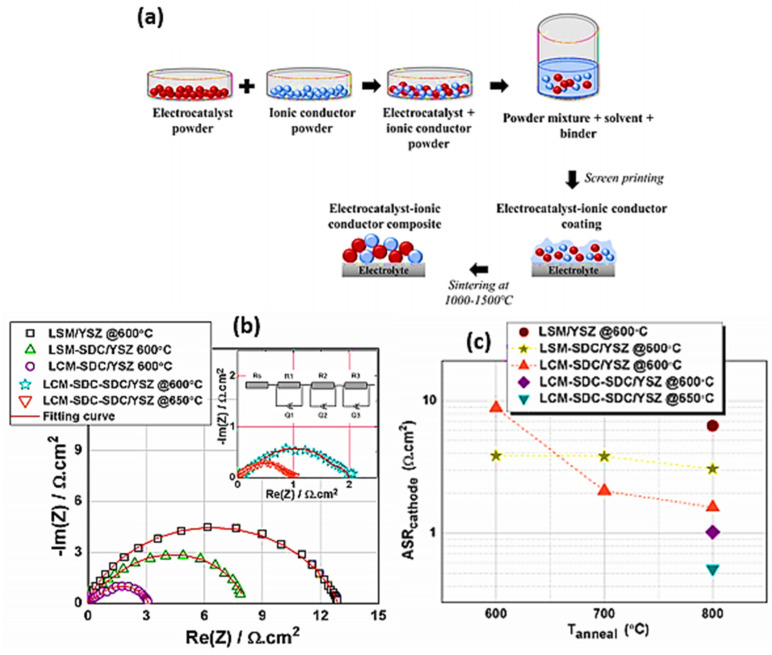
(**a**) Schematic illustration for the fabrication of composite cathode using powder-based precursors. (**b**) The Nyquist plot was obtained for LSM, LSM–SDC, and LCM–SDC thin film on YSZ substrates at 800 °C for 3 h and tested at 600 °C. The inset in (**b**) displays the LCM–SDC thin film on the YSZ substrate with an SDCinterlayer. (**c**) Polarization resistance of the fabricated thin films. Copyright 2019 by American Chemical Society [91].

Microbial fuel cell (MFC) is presently being emphasized as a significant green energy generation device due to its potential to create sustainable energy with little gas emissions [88]. MFC is an electrochemical device that converts the chemical energy of carbohydrates into electricity through the catalytic action of microorganisms in an anaerobic environment [89]. Perovskite cathode materials such as La_0.65_Sr_0.35_MnO_3_ (LSM), La_0.8_Sr_0.2_CoO_3_ (LSC), La_0.6_Sr_0.4_FeO_3_ (LSF) and La_0.6_Sr_0.4_Co_0.2_Fe_0.8_O_3_ (LSCF) were subjected to short-term compatibility tests under air, CO_2_ and air/CO_2_ atmospheres. Moreover, the fabricated nanocomposite fuel cell showed an exceptional performance of 1.06 W cm^−2^ at 550 °C by utilizing the composite, which increases the ionic transport in the electrolyte layer and promotes efficient reaction kinetics at the electrodes [90]. Conventional composite electrodes utilized in SOFCs (solid oxide fuel cells) were made by co-sintering of electrocatalyst with ionic powders at 1100–1250 °C. The SEM and TEM-EDAX studies indicated that La formed clusters with Mn, whereas Ce accumulated as a distinct phase. At all temperatures, the La_0.8_Sr_0.2_MnO_3_-Ce_0.8_Sm_0.2_O_2_ (LSM-SDC) surface accumulated a substantial quantity of Sr, whereas the La_0.8_Ca_0.2_MnO_3_-Ce_0.8_Sm_0.2_O_2_ (LCM-SDC) surface accumulated a small amount of Ca enrichment. When EIS analysis was used to determine the electrochemical activity of the polymeric precursor derived SOFC cathodes, it was observed that the LCM-SDC cathodes had a better ORR activity than the LSM-SDC. With increasing heat treatment temperature in both LSM-SDC and LCM-SDC thin films, cathode resistance decreased, and preferred clustering of cations formed the electrocatalyst and ionic conductor phases (Figure 10a). In the presence of an SDC interlayer at the cathode/electrolyte interface, potential polarization resistances of 0.55Ω cm^2^ at 650 °C were found from LCM-SDC thin films (Figure 10b) [91]. 

**Figure 11:**
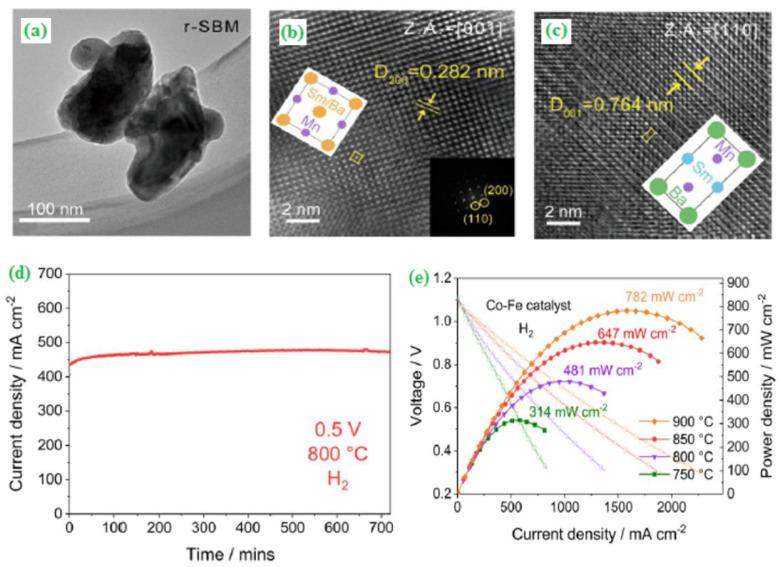
(**a**) TEM image of r–SBM, (**b**) Zone axis of 001 planes, (**c**) Zone axis of 110 planes, (**d**) Short term stability for the symmetrical cell under a constant voltage load of 0.5 V in humidified H_2_ at 800 °C, and (**e**) Current-voltage and power density curves for the symmetrical SBM electrode in the presence of humidified H_2_, anode side infiltrated with 15 wt% Co–Fe catalysts. Copyright 2019 by American Chemical Society [94].

Zhao et al. [92] developed functional nanocomposite electrodes using nanocomposites for the advanced fuel cell technology (NANOCOFC) method that has much potential for improving low-temperature solid oxide fuel cell (LTSOFC) performance utilizing ceria-carbonate nanocomposite electrolytes. For high-performance fuel cells, homogenous, percolating electron and ion phases, as well as well-balanced electronic and ionic conductivities, are all essential. The nanocomposite electrode of LiNiCuZn-NSDC developed by the wet chemical method has excellent potential for high-performance electrodes that are chemically, thermally and mechanically compatible with the samarium doped ceria-carbonate nanocomposite (NSDC) nanocomposite electrolyte. At 550 °C, a maximum 730 mW cm^−2^ was attained in the experiment. In the field of energy storage and conversion, graphene-based nanocomposites have emerged as a new research topic. Graphene has been further changed as a catalyst carrier for hydrogen fuel cells to provide a more excellent uniform metal dispersion, therefore enhancing the electrocatalyst activity [93]. The high-performance-based A-site layered (SmBaMnO_5+δ_, SBM) perovskite has been synthesized by the combustion method. Two different lattice fringes and their corresponding planes of *r*-SBM &*o*-SBM were analyzed by HR-TEM (Figure 11a–c). The as-synthesized layered perovskite (SBM) was a promising candidate, which delivered a maximum power density (782 mW cm^−2^) with satisfactory short-term stability in a symmetrical solid-oxide fuel cell (SSOFC) (Figure 11d,e) [94].

## 4. Nanoscale Based Catalysts for Water Splitting Reaction

There are several possible energy storage methods with electrochemical technology being the most viable and effective way for storing and converting renewable energy. Batteries, flywheels, compressed air, pumped hydroelectricity, electrolysis and magnetic superconductors are all examples of electrochemical technology. Because H_2_ is the ultimate energy carrier, the use of renewable electricity to electrolyze water for the creation of hydrogen is a powerful method of energy storage among the several possible electrochemical energy technologies. As a result, the development of water electrolysis technology for H_2_ generation is necessary and important [95]. In a porous network, free-standing and multi-functional NiSe nanoparticle supported forest carbon nanotube (CNT@NiSe/SS) composite synthesized by the electrodeposition route, due to an easy method, large-scale production and cost-effectiveness. From the XRD pattern, CNT@NiSe/SS has exhibited two different phases, like hexagonal and orthorhombic, respectively. The uniformly distributed and intertwined shell structured morphologically based CNT@NiSe/SS composite were analyzed by TEM with an average diameter of 28 nm. Moreover, the constructed hybrid capacitor (CNT@NiSe/SS and graphene) has a lower overpotential (267 mV vs. RHE) with a high current density (50 mA cm^−2^) and delivered a maximum energy density (32.1 Wh kg^−1^) with a good cyclic stability (floating test up to 50 h) [96]. 

**Figure 12:**
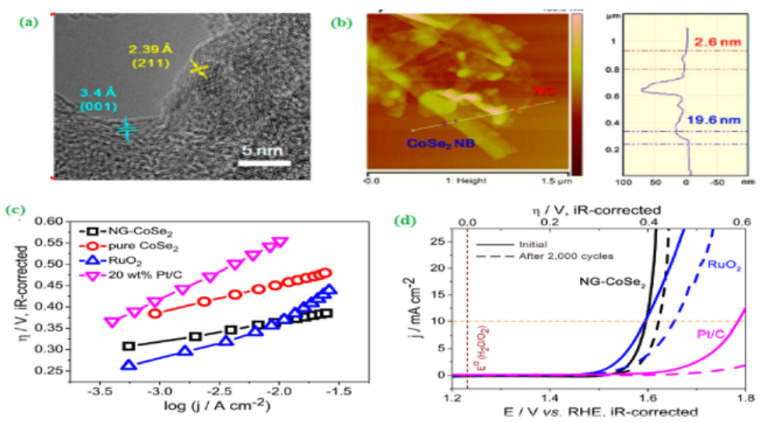
(**a**) HR–TEM image of CoSe_2_ nanobelt, (**b**) AFM image and corresponding height profile of NG-CoSe_2_ composite, (**c**) Tafel plot, and (**d**) OER polarization curves of NGCoSe_2_ composite, RuO_2_, and 20 wt % Pt/C catalysts before and after potential sweeps (0.3–0.8 V vs. Ag/AgCl) for 2000 cycles. Copyright 2014 by American Chemical Society [98].

Qu et al. [97] proposed a three-dimensional (3D) structured CdS/Ni_3_S_2_/PNF nanocomposite for water-splitting applications. The CdS/Ni_3_S_2_/PNF comprises of CdS quantum dots integrated Ni_3_S_2_ nanosheet deposited on the plasma-treated nickel foam (PNF). The fabricated CdS/Ni_3_S_2_/PNF catalyst has a lower onset potential (1.25 V vs. RHE) and superior electrochemical performance for HER and OER activities. Further, the CdS/Ni_3_S_2_/PNF achieved a current density of 10 mA cm^−^^2^ for the HER with a 121 mV overpotential when performing the catalytic reaction in alkaline media. Gao and his co-workers [98] developed a nitrogen-doped graphene-based CoSe_2_ nanobelt (NG-CoSe_2_) composite by the in situ technique. A thin nanobelt morphological structure was examined by HR-TEM (Figure 12a,b). The image revealed that nanoparticles appeared as many tiny clusters. The easily prepared NG-CoSe_2_ catalyst showed excellent OER activity with lower onset potential (η = 0.366V@10 mV cm^−2^) under alkaline conditions (Figure 12c,d). Notably, Qin et al. [99] incorporated N-doped carbon nanotubes with metal oxides to construct a non-noble-metal supported NCNT/MnO-(MnFe)_2_O_3_ tri-functional electrocatalyst by the solvothermal method followed by thermal treatment under both Argon and ammonia atmospheric conditions. The fabricated multi-functional NCNT/MnO-(MnFe)_2_O_3_ catalysts exhibited a high energy density (776 Wh kgzn^−1^), and the assembled catalyst required the potential of about 1.7 V for the water splitting reaction, respectively. In general, a phosphorous-based electrocatalyst can serve as a low-cost electrocatalyst for the water-splitting process. Specifically, a highly stable phosphorous-modified cobalt molybdenum sulfide (P-CoMoS/CC) catalyst displayed outstanding water-splitting performance under alkaline conditions [100]. 

## 5. Electrode Stability

The demand for clean and renewable energy is continuously increasing due to natural disasters and environmental degradation, and therefore, the expansion of potential energy storage and conversion devices has become crucial [101,102,103]. Hydrogen is a prospective sustainable energy source that can be generated by splitting water in electrocatalysis or photocatalysis. Both HER and OER are involved in the electrochemical water-splitting reaction process. Both processes need the use of a catalyst to improve reaction kinetics [104,105,106]. Precious metals (Ru, Pt and Pd) and noble metal oxides (IrO_2_) have shown the most remarkable results in both OER and HER so far. They are, however, hindered in many applications because of their high cost and scarcity [107]. Researchers have been undertaking several efforts to produce non-precious metal-based water-splitting catalysts, such as sulphides [108], selenides [109], and oxides [110].

Carbon/carbonaceous electrode materials have shown outstanding catalytic performances due to their inherent physical properties like chemical and mechanical stability, porosity and pore-size distribution, large surface area, and higher conductivity, compared to other known catalysts [111]. Liu et al. [112] developed an rGO/Si composite utilizing an efficient technique for high-performance lithium-ion battery (LIB) applications. The TEM and EDS analysis revealed uniform distribution of Si nanoparticles on the rGO surface, which can create a 3D network and subsequently increase the electron transport and physical properties of the anode material. Among different ratios of rGO (5, 10 and 15) with Si-600 tested, the 10RGO/Si-600 composite displayed an excellent initial special capacity value of 2317 mA h/g and coulombic efficiency of 93.2% at a current density of 0.1 A/g. After 100 cycles at 2 A/g, the special capacity stays at 728 mA h/g, showing a good cyclic stability. The outstanding electrochemical performance is ascribed to the stable 3D structure, which may significantly influence the buffer volume expansion throughout the cycling process for favourable electrochemical reactions. This research establishes a novel approach for producing improved graphene Si nanocomposites for high-stability LIBs. 

A high-performance Si Np (Silicon Nanoparticle)-based anode was developed by Patil et al. The prepared electrode showed a good sturdy capacity and stable electrochemical cycling. Moreover, it displayed capacity retention of 72% up to the 500th cycle. The electrode has a stable performance which was attributed to the aerogel structure that reduces the needless reactions of the electrolyte with the nanoparticles and forms the stable solid electrolyte interface (SEI) layer [113]. The electrocatalyst Co_2_P was prepared via phosphatization and evaluated its stability in both acidic and alkaline electrolytes for HER applications. The characterization techniques such as electrochemical and spectroscopic methods showed that the Co_2_P degradation has two different paths in acid and alkaline media. Although the Co and P dissolve in acid media, the surface of Co_2_P remained active for HER. In alkaline media, P is specifically dissolved whilst the residual cobalt on the surface converts to hydroxide, thereby decreasing HER activity. The transition metal phosphides (TMPs) developed by various techniques could have diverse dissolution mechanisms. Based on the results obtained, the authors concluded that the *I/V* curve alone does not provide solid evidence to confirm the stability of the electrode. Interestingly, the structural and compositional analysis of electrolyte and electrode revealed that the intrinsic stability and degradation mechanism of the electrocatalysts were reproducible. 

Chung et al. investigated the stability and activity of Au@Pt catalyst by in-situ monitoring of atomic dissolution and physicochemical analysis, and the results were cross-checked with theoretical modelling [114]. The authors pointed out that the stability of Au@Pt system was attributed to the interaction between two metals where Au stabilized Pt by reducing the oxophility of Pt through an electron deficiency-driven ligand effect. The formation of Pt oxide was controlled upon the regulation of the oxophilicity of Pt while Au interacts with Pt. Otherwise, the oxophilicity directly affect the Pt dissolution and causes the catalyst to become less stable. 

**Figure 13:**
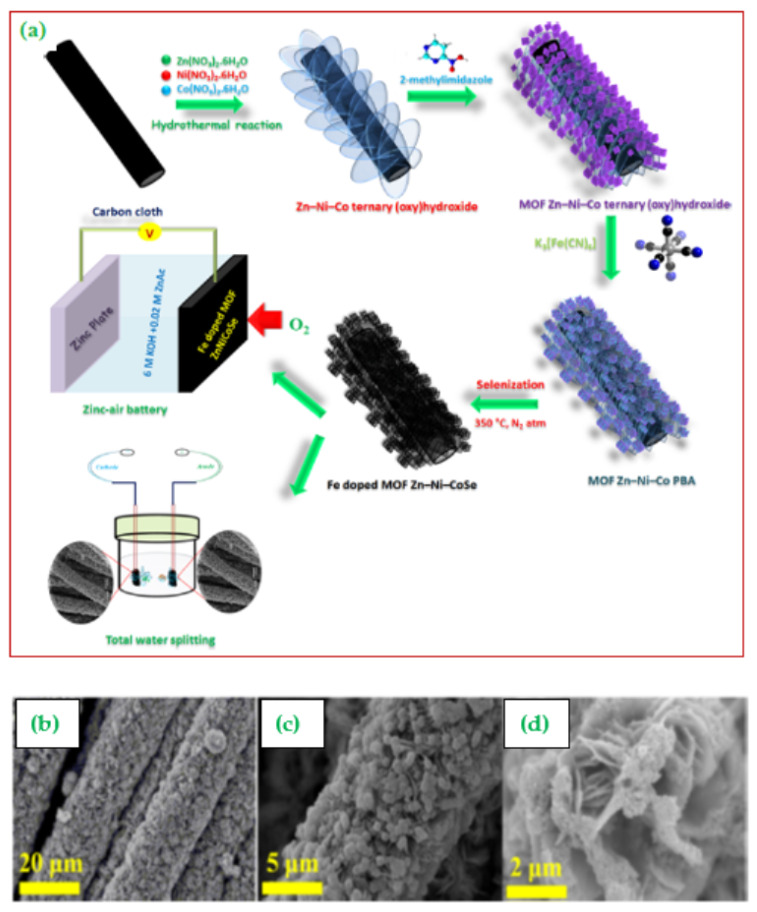
(**a**) Schematic representation of the synthetic strategy and electrochemical applications of Fe–doped MOF ZnNiCoSe@CC, and (**b**–**d**) low- and high-magnification FE–SEM images of Fe–doped MOF ZnNiCoSe@CC. Copyright 2020 by American Chemical Society [115].

Mutharasu et al. [115] constructed a well aligned Fe-doped MOF-framework-based ZnNiCoSe@CC nano-shell catalyst with high surface area and excellent electrical conductivity through the hydrothermal route (Figure 13a). Low and high-magnification FE-SEM images of the developed catalysts are shown in Figure 13b,c. It was found that the hierarchical architecture was extremely porous, while the integration of selenium was due to nanocrystal formation. The developed porous structure renders good mass transport and mechanical stability, which prevents aggregation of 3D domains. Further, the incorporation of Fe can alter the morphological and electronic structure of catalysts.

The as-prepared multifunctional electrocatalyst (MOF ZnNiCoSe@CC) showed superior electrochemical activity (larger capacity) towards OER, ORR and HER with long cycle life (Figure 13b). Interestingly, the Fe-doped MOF ZnNiCoSe@CC catalyst outperforms the conventional Pt/C/RuO2 in the water-splitting reaction. Finally, the developed catalyst was used as an air cathode to construct a rechargeable zinc−air battery which displayed a very impressive discharge−charge activity.

Overall, this review summarized the recent advancement of nanoscale supported electrocatalysts for fuel cell, metal-air battery and water splitting reactions. In addition, we highlighted characterizations, possible mechanisms and various synthetic approaches for the fabrication of advanced nanocomposite electrocatalysts to improve their catalytic activities. Based on the fruitful discussion, morphology, chemical structure, surface area, and electrochemical properties have been optimized through the hydrothermal route. Finally, the literature emphasizes that nanocomposite-based electrocatalysts have a more promising architecture to achieve their electrochemical properties and provide new sources for the production of large-scale practical applications in the future.

## Data Availability

Not applicable.

## Supplementary Materials

The following supporting information can be downloaded at: www.mdpi.com/article/10.3390/ma15020458/s1, Table S1: Comparison of fuel cell performances on various electrocatalysts.; Table S2: Summary of ORR and OER electrocatalytic activities of the reported various types of advanced nanoscale based catalysts.
