# Supplementary material for: Recent Advances in Nanoscale Based Electrocatalysts for Metal-Air Battery, Fuel Cell and Water-Splitting Applications: An Overview
Source: Materials (Basel). 2022 Jan 8;15(2):458. doi: 10.3390/ma15020458 (PMC8778511; doi:10.3390/ma15020458)
Supplement: Supplementary file 1 [file materials-15-00458-s001.zip › materials-1458647-supplementary.pdf]

materials

MDPI

Supplementary Information

Recent Advances in Nanoscale Based Electrocatalysts for Metal-Air Battery, Fuel Cell and Water-Splitting Applications: An Overview

Tse-Wei Chen [1], Ganesan Anushya [2], Shen-Ming Chen [3,*], Palraj Kalimuthu [4], Vinitha Mariyappan [3], Pandi Gajendran [5] and Rasu Ramachandran [5,*]

1 Department of Materials, Imperial College London, London SW7 2AZ, UK; t.chen19@imperial.ac.uk
2 Department of Physics, S.A.V. Sahaya Thai Arts and Science (Women) College, Sahayam Nagar, Kumarapuram Road, Vadakkankulam, Tirunelveli 627116, India; anushya@savsahayathaicollege.com
3 Electroanalysis and Bioelectrochemistry Laboratory., Department of Chemical Engineering and Biotechnology, National Taipei University of Technology, No.1, Section 3, Chung-Hsiao East Road, Taipei 106, Taiwan; vinithavicky80@gmail.com
4 School of Chemistry and Molecular Biosciences, University of Queensland, Brisbane 4072, Australia; p.kalimuthu@uq.edu.au
5 Department of Chemistry, The Madura College, Vidya Nagar, Madurai 625011, India; haigaja78@yahoo.com
* Correspondence: smchen@ntut.edu.tw (S.-M.C.); ramachandran@maduracollege.edu.in (R.R.)

Citation: Chen, T.-W.; Anushya, G.; Chen, S.-M.; Kalimuthu, P.; Mariyappan, V.; Gajendran, P.; Ramachandran, R. Recent Advances in Nanoscale Based Electrocatalysts for Metal-Air Battery, Fuel Cell and Water-Splitting Applications; An Overview. *Materials* **2022**, *15*, 458. https://doi.org/10.3390/ma15020458

Academic Editors: Vincenzo Baglio and Carmelo Lo Vecchio

Received: 27 October 2021
Accepted: 4 January 2022
Published: 8 January 2022

Publisher's Note: MDPI stays neutral with regard to jurisdictional claims in published maps and institutional affiliations.

Copyright: © 2022 by the authors. Submitted for possible open access publication under the terms and conditions of the Creative Commons Attribution (CC BY) license (https://creativecommons.org/licenses/by/4.0/).

Table S1. Comparison of fuel cell performances on various electrocatalysts.

Synthesis Route	Catalysts	Overpotential/Onset potential (mV)	Current Density (mA cm^{-2})	Medium (M)	Ref.
Sol-gel	Pt/Clay/Nafion	910	4.2	05 H_2SO_4	[1]
Solvothermal	PVA/SGO/Fe_3O_4	670	25.57	1M NaCl	[2]
Hummer's	Fe/Ni-MOFs/NG-20	109	8.56	0.1 KOH	[3]
Calcinations	Hematite-LCP electrolyte	940	-	$LaCePrO_x$ (LCP)	[4]
CVD	Pt/GC-240<br>Pt/GC-500	940<br>940	661<br>653	Polyelectrolyte	[5]
Hydrothermal	Pt/TiO_2/rGO	750	45.8	05 H_2SO_4	[6]
Hummer's	Pt-MnO_2-ERGO	360	1.76	01 H_2SO_4	[7]
Hummer's	XC-72/Carbon/Pd	550	7.3	05 H_2SO_4	[8]
Electrodeposition	Co_3O_4-3DrGO/Nif	442	0.012	0.1 KOH	[9]
Solution casting	SPEEK/PABS-SWCNT	660	80	05 H_2SO_4	[10]

Table S2. Summary of ORR and OER electrocatalytic activities of the reported various types of advanced nanoscale based catalysts.

Synthesis Route	Catalysts	Morphology	Specific Capacity (mAh g^{-1})	Current Density (mA cm^{-2})	Medium (M)	Ref.
Electrospinning	Fe_3C@N-CFs	Nanofiber	1287.3	10	0.1 KOH	[11]
Coating	Co^{II}-salen/MWCNT@MnO_2	Nanosheet	1000	0.15	-	[12]
Electrodeposition	Co_3O_4@Co/NCNT	Core-shell	891	135	0.5 H2SO4	[13]
Hydrothermal	Zn-Co-SNN/CFP	Nanoplate	484.7	10	0.1 KOH	[14]
Hydrothermal	NP Co_3O_4/Fe@C_2N	Nanoparticles	790.1	165.8	0.1 KOH	[15]
Hydrothermal	800N,P-CNT	Porous structure	762	20	0.1 KOH	[16]
Solid-state	PANI@ZnPC	Pores structure	713	-	7 KOH	[17]
Dissolution	PVA-5wt.%SiO_2GPE	Porous structure	720.6	0.3	0.1 KOH	[18]
Hydrothermal	8-MnO_2/N-rGO	Wrinkled structure	5250	0.2	0.1 KOH	[19]
Solution combustion	Co/$CoFe_2O_4$	Porous structure	4320	100	0.1 KOH	[20]

References

1. Qin, X.; Huang, Y.; Wang, K.; Xu, T.; Wang, Y.; Wang, M.; Zhao, M.; Gao, Q. High efficient oxygen reduction reaction catalyst derived from Fe/Ni-mixed metal-organic frame works for application of fuel cell cathode. *Ind. Eng. Chem. Res*. **2019**, *58*, 10224–10237.
2. Narayanamoorthy, B.; Datta, K.K.R.; Eswaramoorthy, M.; Balaji, S. Improved Oxygen Reduction Reaction Catalyzed by Pt/Clay/Nafion Nanocomposite for PEM Fuel Cells. *ACS Appl. Mater. Interfaces* **2012**, *4*, 3620–3626, doi:10.1021/am300697q.
3. Beydaghi, H.; Javankakht, M. Aligned nanocomposite membrane containing sulfonated graphene oxide with superior ionic conductivity for direct methanol fuel cell applications. *Ind. Eng. Chem. Res*. **2015**, *54*, 7028–7037.
4. Xia, C.; Cai, Y.; Ma, Y.; Wang, B.; Zhang, W.; Karlsson, M.; Wu, Y.; Zhu, B. Natural Mineral-Based Solid Oxide Fuel Cell with Heterogeneous Nanocomposite Derived from Hematite and Rare-Earth Minerals. *ACS Appl. Mater. Interfaces* **2016**, *8*, 20748–20755, doi:10.1021/acsami.6b05694.
5. Ramaprabhu, S.; Aravind, S.S.J. Pt-nanoparticle-dispersed graphene-wrapped MWCNT composites as oxygen reduction electrocatalyst in proton exchange membrane fuel cell. *ACS Appl. Mater. Interfaces* **2012**, *4*, 3805–3810.
6. Xia, B.Y.; Wu, H.B.; Chen, J.S.; Wang, Z.; Wang, X.; Lou, X.W. Formation of Pt-TiO_2-rGO_3-phase junction with significantly enhanced electro-activity for methanol oxidation. *Phys. Chem. Chem. Phys*. **2012**, *14*, 473–476.
7. Ezhilvilian, A.T.; Rajkumar, M.; Chen, S.M.; Hu, C.C.; Boopathi, K.M.; Chu, C.W. Highly electrocatalytic performance of platinum and manganese dioxide nanoparticle decorated reduced graphene oxide sheets for methanol electro-oxidation. *RSC Adv*. **2014**, *4*, 41387–41397.
8. Huang, H.; Wang, X. Pd nanoparticles supported on low-defect graphene sheets: for use as high-performance electrocatalysts for formic acid and methanol oxidation. *J. Mater. Chem*. **2012**, *22*, 22533–22541, doi:10.1039/c2jm33727d.
9. Sundardey, R.; Purkait, T. Fabrication of a membraneless non-enzymatic glucose-air fuel cell with graphene-cobalt oxide nanocomposite anode and Fe, N-doped biomass carbon cathode. J.Electroanal.Chem. 2020, 874, 114467.
10. Shukla, A.; Dhanasekaran, P.; Sasikala, S.; Nagaraju, N.; Bhat, S.D.; Pillai, V.K. Nanocomposite membrane electrolyte of polyamino benzene sulfonic acid grafted single walled carbon nanotubes with sulfonated polyether ketone for direct methanol fuel cells. *Int. J. Hydrogen Energy*. **2019**, *44*, 27564–27574, doi:10.1016/j.ijhydene.2019.08.189.
11. Ma, Y.; Sumboja, A.; Zhang, W.; Yin, S.; Wang, S.X.; Pennycook, S.J.; Kou, Z.; Li, X.; Wang, J. Flexible and warable all-solid-state Al-air battery based on iron carbide encapsulated in electrospun porous carbon nanofibers. *ACS Appl. Mater Interfaces* **2019**, *11*, 1988–1995.
12. Hu, X.; Wang, J.; Li, X.; Wang, J.; Gregory, D.H.; Chen, J. MWCNT@MnO_2 nanocomposite cathode integrated with soluble O-carrier Co-salen in electrolyte for high-performance Li-Air batteries. *Nano Lett*. **2017**, *17*, 2073–2078.
13. Sing, T.; Das, C.; Bothra, N.; Sikdar, N.; Das, S.; Pati, S.K.; Maji, T.K. MOF derived Co_3O_4Co/NCNT nanocomposite for electrochemical hydrogen evolution, flexible Zinc-air batteries and overall water splitting. *Inorg. Chem*. **2020**, *5*, 3160–3170.
14. Wu, X.; Han, X.; Ma, X.; Zhang, W.; Deng, Y.; Zhong, C.; Hu, W. Morphology-controllable synthesis of Zn-Co mixed sulfide

nanostructures on carbon fiber paper towards efficient rechargeable Zinc-air batteries and water electrolysis. *ACS Appl. Mater. Interfaces* **2017**, *9*, 12574–12583.

15. Kim, J.; Gwon, O.; Mahmood, J.; Kim, C.; Yang, Y.; Lee, H.; Lee, J.H.; Jeong, H.Y.; Baek, J.B.; Kim, G. Synergetic coupling derived cobalt oxide with nitrogenated holly two-dimensional matrix as an efficient bifunctional catalyst for metal-air batteries. *ACS Nano.* **2019**, *5*, 5502–5512.
16. Li, Z.; Zhao, W.; Yin, C.; Wei, L.; Wu, W.; Hu, Z.; Wu, M.; Synergistic effects between doped nitrogen and phosphorous in metal-free cathode for Zinc-air battery from covalent organic frameworks coated CNT. *ACS Appl. Mater. Interfaces* **2017**, *9*, 44519–44528.
17. Deyab, M.; Mele, G.; Protected], [Email Polyaniline/Zn-phthalocyanines nanocomposite for protecting zinc electrode in Zn-air battery. *J. Power Sources* **2019**, *443*, 227264, doi:10.1016/j.jpowsour.2019.227264.
18. Fan, X.; Liu, J.; Song, Z.; Han, X.; Deng, Y.; Zhong, C.; Hu, W. Porous nanocomposite gel polymer electrolyte with high ionic conductivity and superior electrolytic retention capacity for long-cycle life flexible Zinc-air batteries. Nano Energy, 56, 454–462.
19. Zhoor, A.; Faizan, R.; Elsaid, K.; Hashmi, S.; Butt, F.A.; Ghouri, Z.H. Synthesis and experimental investigation of δ-MnO_2/N-rGO nanocomposite for Li-O2 batteries applications. *Chem. Eng. J. Adv.* **2021**, *7*, 100115.
20. Athika, M.; Elumalai, P. Co/$CoFe_2O_4$ decorated porous carbon network as air-breathing electrode for high-capacity rechargeable lithium-air battery; Role of Metallic Co nanoparticle. *Chem. Electro. Chem.* **2020**, *7*, 4188–4200.
